# Threshold Dynamics in Stochastic SIRS Epidemic Models with Nonlinear Incidence and Vaccination

**DOI:** 10.1155/2017/7294761

**Published:** 2017-01-16

**Authors:** Lei Wang, Zhidong Teng, Tingting Tang, Zhiming Li

**Affiliations:** ^1^Department of Medical Engineering and Technology, Xinjiang Medical University, Urumqi 830011, China; ^2^College of Mathematics and System Sciences, Xinjiang University, Urumqi 830046, China

## Abstract

In this paper, the dynamical behaviors for a stochastic SIRS epidemic model with nonlinear incidence and vaccination are investigated. In the models, the disease transmission coefficient and the removal rates are all affected by noise. Some new basic properties of the models are found. Applying these properties, we establish a series of new threshold conditions on the stochastically exponential extinction, stochastic persistence, and permanence in the mean of the disease with probability one for the models. Furthermore, we obtain a sufficient condition on the existence of unique stationary distribution for the model. Finally, a series of numerical examples are introduced to illustrate our main theoretical results and some conjectures are further proposed.

## 1. Introduction

As is well known, transmissions of many infectious diseases are inevitably affected by environment white noise, which is an important component in realism, because it can provide some additional degrees of realism compared to their deterministic counterparts. Therefore, in recent years, stochastic differential equation (SDE) has been used widely by many researchers to model the dynamics of spread of infectious disease (see [1–5] and the references cited therein). There are different possible approaches to include effects in the model. Here, we mainly introduce three approaches. The first one is through time Markov chain model to consider environment noise in SIS model (see, e.g., [6] and the references cited therein). The second is with parameters perturbation (see [2, 5, 7] and the references cited therein). The last issue to model stochastic epidemic system is to perturb around the positive equilibria of deterministic models (see, e.g., [1, 8, 9] and the references cited therein).

Now, we consider stochastic epidemic models with parameters perturbation. The incidence rate of a disease denotes the number of new cases per unit time, and this plays an important role in the study of mathematical epidemiology. In many epidemic models, the bilinear incidence rate *βSI* is frequently used (see [2, 5, 7, 8, 10–17]), and the saturated incidence rate *βSI*/(1 + *aI*) is also frequently used (see, e.g., [18–22]). Comparing with bilinear incidence rate and saturated incidence rate, Lahrouz and Omari [23] and Liu and Chen [24] introduced a nonlinear incidence rate *βSI*/*φ*(*I*) into stochastic SIRS epidemic models. In [25], Tang et al. investigated a class of stochastic SIRS epidemic models with nonlinear incidence rate *βf*(*S*)*g*(*I*):

(1) dS=Λ−βfSgI−μS+δRdt−σfSgIdBt,dI=βfSgI−μ+α+γIdt+σfSgIdBt,dR=γI−μ+δRdt.

Lahrouz et al. [26] studied a deterministic SIRS epidemic model with nonlinear incidence rate *βSI*/*φ*(*I*) and vaccination. If the transmission of the disease is changed by nonlinear incidence rate *βf*(*S*)*g*(*I*), and to make the model more realistic, let us suppose that the death rates of the three classes in the population are different, then a more general deterministic SIRS model is described by the following ordinary differential equation:

(2) S˙=1−qΛ−βfSgI−dS+pS+εR,I˙=βfSgI−dI+γI,R˙=qΛ+pS+γI−dR+εR,

where *S*(*t*), *I*(*t*), and *R*(*t*) denote the numbers of susceptible, infectious, and recovered individuals at time *t*, respectively. Λ denote a constant input of new members into the susceptible per unit time. *q* is the rate of vaccination for the new members. *p* is the rate of vaccination for the susceptible individuals. *d*_*S*_ is the natural mortality rate or the removal rate of the *S*. *d*_*I*_ is the removal rate of the infectious and usually is the sum of natural mortality rate and disease-induced mortality rate. *d*_*R*_ is the removal rate of the recovered individual. *γ* is the recovery rate of infective individual. *ε* is the rate at which the recovered individual loses immunity. *β* represents the transmission coefficient between compartments *S* and *I*, and *βf*(*S*)*g*(*I*) denotes the incidence rate of the disease. For biological reasons, we usually assume that functions *f*(*S*) and *g*(*I*) satisfy the following properties:

(**H**_**1**_) *g*(*I*) is two-order continuously differentiable function; *g*(*I*)/*I* is monotonically nondecreasing with respect to *I*; *g*(0) = 0 and *g*′(0) > 0.
 (**H**_**2**_) *f*(*S*) is two-order continuously differentiable function; *f*′(*S*) ≥ 0 and *f*′′(*S*) ≤ 0 for all *S* ≥ 0, and *f*(0) = 0.

It is well known that the basic reproduction number for model (2) is defined by *R*_0_ = *βf*(*S*_0_)*g*′(0)/(*d*_*I*_ + *γ*), where *S*_0_ = [(1 − *q*)*d*_*R*_ + *ε*]Λ/(*d*_*S*_(*d*_*R*_ + *ε*) + *pd*_*R*_). Applying the Lyapunov function method and the theory of persistence for dynamical systems, we can prove that, when *R*_0_ < 1, model (2) has a globally asymptotically stable disease-free equilibrium *E*_0_ = (*S*_0_, 0, *R*_0_) and, when *R*_0_ > 1, model (2) has a unique endemic equilibrium *E*^*∗*^(*S*^*∗*^, *I*^*∗*^, *R*^*∗*^) and disease *I* is permanent.

In this paper, we extend model (1) to more general cases. As in [11], taking into account the effect of randomly fluctuating environment, we assume that fluctuations in the environment will manifest themselves mainly as fluctuations in parameters *β*, *d*_*S*_, *d*_*I*_, and *d*_*R*_ in model (2) change to random variables $\tilde{\beta }$, ${\tilde{d}}_{S}$, ${\tilde{d}}_{I}$, and ${\tilde{d}}_{R}$ such that

(3) β~=β+error0,d~S=dS+error1,d~I=dI+error2,d~R=dR+error3.

Accordingly, model (2) becomes

(4) dS=1−qΛ−βfSgI−dS+pS+εRdt−fSgIerror0 dt−S error1 dt,dI=βfSgI−dI+γIdt+fSgIerror0 dt−I error2 dt,dR=qΛ+pS+γI−dR+εRdt−R error3 dt.

By the central limit theorem, the error term error_*i*_ *dt* (0 ≤ *i* ≤ 3) may be approximated by a normal distribution with zero mean and variance *σ*_*i*_^2^*dt* (0 ≤ *i* ≤ 3), respectively. That is, ${error}_{i}\;dt=\tilde{N}(0,\sigma_{i}^{2}dt)$. Since these error_*i*_ *dt* may correlate with each other, we represent them by *l*-dimensional Brownian motion *B*(*t*) = (*B*_1_(*t*),…, *B*_*l*_(*t*)) as follows:

(5) $$\begin{array}{c} {error}_{i}\;dt=\overset{l}{\underset{j=1}{\sum }}\sigma_{ij}dB_{j}\left(\;t\;\right),\;0\leq i\leq 3, \end{array}$$

where *σ*_*ij*_ are all nonnegative real numbers. Therefore, model (4) is characterized by the following Itô stochastic differential equation:

(6) dS=1−qΛ−βfSgI−dS+PS+εRdt−fSgI∑j=1lσ0jdBjt−S∑j=1lσ1jdBjt,dI=βfSgI−dI+γIdt+fSgI∑j=1lσ0jdBjt−I∑j=1lσ2jdBjt,dR=qΛ+pS+γI−dR+εRdt−R∑j=1lσ3jdBjt.

Model (6) in the special case where *f*(*S*) = *S*, *g*(*I*) = *I*, and *p* = *q* = 0 has been investigated by Yang and Mao in [11] and in the special case where *σ*_1*j*_ = *σ*_2*j*_ = *σ*_3*j*_ = 0  (1 ≤ *j* ≤ *l*) and *p* = *q* = 0 also has been discussed in [25]. It is well known that, in a stochastic epidemic model, the dynamical behaviors, like the extinction, persistence, stationary distribution, and stability of the model, are the most interesting topics. Therefore, in this paper, as an important extension and improvement of the results given in [11, 25], we aim to discuss the dynamical behaviors of model (6). Particularly, we will explore the stochastic extinction and persistence in the mean of disease with probability one and the existence of stationary distribution.

This paper is organized as follows. In Section 2, we introduce some preliminaries to be used in later sections. In Section 3, we establish the threshold condition for stochastic extinction of disease with probability one of model (6). In Section 4, we deduce the threshold conditions for the disease being stochastically persistent and permanent in the mean with probability one. In Section 5, we discuss the existence of the stationary distribution of model (6) under some sufficient conditions. In Section 6, the numerical simulations are presented to illustrate the main results obtained in this paper and some conjectures are further proposed. Finally, in Section 7, a brief conclusion is given.

## 2. Preliminaries

Through this paper, we let (*Ω*, *ℱ*, {*ℱ*_*t*_}_*t*≥0_, *P*) be a complete probability space with a filtration {*ℱ*_*t*_}_*t*≥0_ satisfying the usual conditions (that is, it is right continuous and increasing while *ℱ*_0_ contains all *P*-null sets). In this paper, we always assume that stochastic model (6) is defined on probability space (*Ω*, *ℱ*, {*ℱ*_*t*_}_*t*≥0_, *P*). Furthermore, we denote *R*_+_^3^ = {(*x*, *y*, *z*) : *x* > 0, *y* > 0, *z* > 0}, *σ*_*i*_^2^ = ∑_*j*=1_^*l*^*σ*_*ij*_^2^, 0 ≤ *i* ≤ 3, and *σ*^2^ = ∑_*i*=0_^3^*σ*_*i*_^2^.

Firstly, on the existence and uniqueness of global positive solution for model (4) we have the following result.

**Lemma 1 .**

Assume that (**H**_1_) and (**H**_2_) hold; then, for any initial value (*S*(0), *I*(0), *R*(0)) ∈ *R*_+_^3^, model (6) has a unique solution (*S*(*t*), *I*(*t*), *R*(*t*)) defined for all *t* ≥ 0, and the solution will remain in *R*_+_^3^ with probability one.

This lemma can be proved by using a similar argument as in the proof of Theorem 3.1 given in [11]. We hence omit it here.

**Lemma 2 .**

Assume that (**H**_1_) and (**H**_2_) hold and let (*S*(*t*), *I*(*t*), *R*(*t*)) be the solution of model (6) with initial value (*S*(0), *I*(0), *R*(0)) ∈ *R*_+_^3^. Then lim sup_*t*→*∞*_(*S*(*t*) + *I*(*t*) + *R*(*t*)) < *∞*  *a*.*s*. Moreover, let *h*(*x*, *y*, *z*) be any continuous function defined on *R*_+_^3^; then for each 1 ≤ *j* ≤ *l* we have

(7) $$\begin{array}{c} \underset{t\to \infty }{lim}\frac{1}{t}\overset{t}{\underset{0}{\int }}h\left(\;S\left(\;s\;\right),I\left(\;s\;\right),R\left(\;s\;\right)\;\right)dB_{j}\left(s\right)=0. \end{array}$$

**Proof**

Let *N*(*t*) = *S*(*t*) + *I*(*t*) + *R*(*t*); then we have from model (6)

(8) dNtΛ−dSSt−dIIt−dRRtdt−Pt≤Λ−μNtdt−Pt,

where *μ* = min {*d*_*S*_, *d*_*I*_, *d*_*R*_} and *P*(*t*) = ∑_*j*=1_^*l*^(*σ*_1*j*_*S*(*t*) + *σ*_2*j*_*I*(*t*) + *σ*_3*j*_*R*(*t*))*dB*_*j*_(*t*). By the comparison theorem of stochastic differential equations, we further have

(9) $$\begin{array}{c} N\left(\;t\;\right)\leq N\left(\;0\;\right)e^{-\mu t}+\frac{\Lambda }{\mu }\left(\;1-e^{-\mu t}\;\right)-Q\left(\;t\;\right), \end{array}$$

where

(10) Qt=∑j=1l∫0te−μt−sσ1jSs+σ2jIs+σ3jRsdBjs.

Define *X*(*t*) = *N*(0) + *A*(*t*) − *U*(*t*) − *Q*(*t*), where *A*(*t*) = (Λ/*μ*)(1 − *e*^−*μt*^) and *U*(*t*) = *N*(0)(1 − *e*^−*μt*^). It is clear that from Lemma 1 and (9) *X*(*t*) is nonnegative for *t* ≥ 0, and *A*(*t*) and *U*(*t*) are continuous adapted increasing processes for *t* ≥ 0 and *A*(0) = *U*(0) = 0. Therefore, by Theorem 3.9 given in [27], we obtain that lim_*t*→*∞*_*X*(*t*) < *∞*  a.s. exists. From (9), we further have

(11) $$\begin{array}{c} \underset{t\to \infty }{\mathrm{limsup}}\;N\left(\;t\;\right)<\infty \text{ a.s}. \end{array}$$

Denote

(12) $$\begin{array}{c} M_{j}\left(\;t\;\right)=\overset{t}{\underset{0}{\int }}h\left(\;S\left(\;s\;\right),I\left(\;s\;\right),R\left(\;s\;\right)\;\right)dB_{j}\left(s\right). \end{array}$$

By (11), we have

(13) 1tMj,Mjt1t∫0thSs,Is,Rs2ds≤supt≥0hSt,It,Rt2<∞.

Hence, the strong law of large number (see [27, 28]) implies lim_*t*→*∞*_(1/*t*)*M*_*j*_(*t*) = 0  a.s. This completes the proof.

For any function *h*(*t*) defined on *R*_+0_ = [0, +*∞*), we denote the average value on [0, *t*] by 〈*h*(*t*)〉 = (1/*t*)∫_0_^*t*^*h*(*s*)*ds*.

**Lemma 3 .**

Assume that (**H**_1_) and (**H**_2_) hold. Let (*S*(*t*), *I*(*t*), *R*(*t*)) be any positive solution of model (6); then

(14) St=1−qdR+εΛdSdR+ε+pdR−dIdR+ε+dRγdSdR+ε+pdRIt+φt,

where function *φ*(*t*) is defined for all *t* ≥ 0 satisfying lim_*t*→*∞*_*φ*(*t*) = 0.

**Proof**

Taking integration from 0 to *t* for model (6), we get

(15) St−S0t=1−qΛ−βStgIt−dS+pSt+εRt−1t∫0t∑j=1lSsgIsσ0j+Ssσ1jdBjs,It−I0t=βStgIt−dI+γIt+1t∫0t∑j=1lSsgIsσ0j−Isσ2jdBjs,Rt−R0t=qΛ+γIt+pSt−dR+εRt−1t∫0t∑j=1lRsσ3jdBjs.

Hence, we have

(16) dR+εSt−S0t+It−I0t+ε·Rt−R0t=1−qdR+εΛ−dSdR+ε+pdRSt−dIdR+ε+dRγIt−1t·∫0t∑j=1NdR+εSsσ1j+Isσ2j+εRsσ3jdBjs.

With a simple calculation from (16) we can easily obtain formula (14) with which *φ*(*t*) is defined by

(17) φt=−1dSdR+ε+pdRdR+ε·St−S0t+It−I0t+εRt−R0t+1t·∫0t∑j=1ldR+εSsσ1j+Isσ2j+εRsσ3jdBjs.

By Lemma 2, we further have lim_*t*→*∞*_*φ*(*t*) = 0  a.s.

**Lemma 4 .**

Assume that (**H**_1_) and (**H**_2_) hold and *σ*_1*j*_ = *σ*_2*j*_ = *σ*_3*j*_ = 0  (1 ≤ *j* ≤ *l*). Then, for any solution (*S*(*t*), *I*(*t*), *R*(*t*)) of system (6) with (*S*(0), *I*(0), *R*(0)) ∈ *R*_+_^3^, one has

(18) $$\begin{array}{c} \underset{t\to \infty }{\mathrm{limsup}}\left(\;S\left(\;t\;\right)+I\left(\;t\;\right)+R\left(\;t\;\right)\;\right)\leq \bar{S},\text{ a.s.}, \end{array}$$

where $\bar{S}=\Lambda /\mu$. Furthermore, the region

(19) $$\begin{array}{c} \Gamma =\left\{\;\left(\;S,I,R\;\right):S>0,I>0,R>0,S+I+R\leq \bar{S}\text{ a.s.}\;\right\} \end{array}$$

is positive invariant with probability one for model (6), where *μ* = min⁡{*d*_*S*_, *d*_*I*_, *d*_*R*_}.

In fact, for *N*(*t*) = *S*(*t*) + *I*(*t*) + *R*(*t*), from model (6) we have

(20) dNtΛ−dSSt−dIIt−dRRtdt≤Λ−μNtdt,  a.s.

This implies that (18) holds, and set Γ is positive invariant with probability one for model (6).

**Lemma 5 .**

Assume that (**H**_1_) and (**H**_2_) hold, *σ*_1*j*_ = *σ*_2*j*_ = *σ*_3*j*_ = 0  (1 ≤ *j* ≤ *l*), *d*_*S*_ = *d*_*R*_, and *d*_*I*_ = *d*_*S*_ + *α* with constant *α* ≥ 0. Then, for any solution (*S*(*t*), *I*(*t*), *R*(*t*)) of model (6) with (*S*(0), *I*(0), *R*(0)) ∈ *R*_+_^3^, one has

(21) St=Λ1−qdS+εdSdS+ε+p+Ht+Gt,  a.s.∀t≥0,

where

(22) Ht=pΛ1−qdS+εdSdS+εdS+ε+pe−dS+ε+pt−R0−qΛdS+ε1p1−e−pte−dS+εt+ΛdS−S0+I0+R0·1p+ε1−e−p+εte−dSt,

(23) Gt=−It−α∫0te−dSt−sIsds−γ∫0te−dS+εt−sIsds+p∫0te−dS+ε+pt−sIsds+pα∫0te−dS+ε+pt−s·∫0se−dSs−uIudu ds+pγ∫0te−dS+ε+pt−s·∫0se−dS+εs−uIudu ds.

**Proof**

Since

(24) $$\begin{array}{c} dN\left(\;t\;\right)=\left(\;\Lambda -d_{S}N\left(\;t\;\right)-\alpha I\left(\;t\;\right)\;\right)dt,\text{ a.s.}, \end{array}$$

then

(25) Nt=ΛdS+N0−ΛdSe−dSt−α∫0te−dSt−sIsds,  a.s.,

where *N*(0) = *S*(0) + *I*(0) + *R*(0). From the third equation of model (6) we have

(26) Rt=qΛdS+ε+R0−qΛdS+εe−dS+εt+p∫0te−dS+εt−sSsds+γ∫0te−dS+εt−sIsds,  a.s.

Therefore,

(27) St=Λ1−qdS+εdSdS+ε−It−R0−qΛdS+εe−dS+εt+N0−ΛdSe−dSt−p∫0te−dS+εt−sSsds−α∫0te−dSt−sIsds−γ∫0te−dS+εt−sIsds.

Let *y*(*t*) = ∫_0_^*t*^*e*^(*d*_*S*_ + *ε*)*s*^*S*(*s*)*ds*; then

(28) dyt=edS+εtStdt=−pyt+Λ1−qdS+εdSdS+εedS+εt−ItedS+εt−R0−qΛdS+ε+N0−ΛdSeεt−αeεt∫0tedSsIsds−γ∫0tedS+εsIsdsdt.

Solving *y*(*t*), we obtain

(29) yt=e−ptΛ1−qdS+εdSdS+εdS+ε+pedS+ε+pt−1−∫0tedS+ε+psIsds−R0−qΛdS+ε1pept−1+N0−ΛdS1ε+peε+pt−1−γ∫0teps∫0sedS+εuIudu ds−α∫0tedS+ε+ps∫0se−dSs−uIuds dsdt.

Substituting (29) into (27), we immediately obtain (21)–(23). This completes the proof.

**Remark 6 .**

When *d*_*S*_ ≠ *d*_*R*_ in model (6), whether we can also establish a similar result as in Lemma 5 still is an interesting open problem.

Consider the following *n*-dimensional stochastic differential equation:

(30) $$\begin{array}{c} dx\left(\;t\;\right)=b\left(\;x\;\right)dt+\overset{m}{\underset{r=1}{\sum }}\sigma_{r}\left(\;x\;\right)dB_{r}\left(t\right), \end{array}$$

where *x* = (*x*_1_, *x*_2_,…, *x*_*n*_), *σ*_*r*_(*x*) = (*σ*_*r*_^1^(*x*), *σ*_*r*_^2^(*x*),…, *σ*_*r*_^*n*^(*x*)), and *B*_*r*_(*t*)  (1 ≤ *r* ≤ *m*) are standard Brownian motions defined on the above probability space. The diffusion matrix is defined by

(31) Ax=aijxn×n,aijx=∑r=1mσrixσrjx.

For any second-order continuously differentiable function *V*(*x*), we define

(32) $$\begin{array}{c} LV\left(\;x\;\right)=\overset{n}{\underset{i=1}{\sum }}\frac{\partial V\left(x\right)}{\partial x_{i}}b_{i}\left(\;x\;\right)+\frac{1}{2}\overset{n}{\underset{i,j=1}{\sum }}\frac{\partial^{2}V\left(x\right)}{\partial x_{i}\partial x_{j}}a_{ij}\left(\;x\;\right). \end{array}$$

The following lemma gives a criterion for the existence of stationary distribution in terms of Lyapunov function.

**Lemma 7 (see [27]).**

Assume that there is a bounded open subset *D* in *R*^*n*^ with a regular (i.e., smooth) boundary such that

(i) there exist some *i* = 1,2,…, *n* and positive constant *η* > 0 such that *a*_*ii*_(*x*) ≥ *η* for all *x* ∈ *D*;
(ii) there exists a nonnegative function *V*(*x*) : *D*^*c*^ → *R* such that *V*(*x*) is second-order continuously differentiable function and that, for some *θ* > 0, *LV*(*x*)≤−*θ* for all *x* ∈ *D*^*c*^, where *D*^*c*^ = *R*^*n*^∖*D*.

Then (30) has a unique stationary distribution *π*. That is, if function *f* is integrable with respect to the measure *π*, then for all *x*_0_ ∈ *R*^*n*^

(33) Plimt→∞1t∫0tfxu,x0du=∫Rnfx0πdx0=1.

To study the permanence in mean with probability one of model (6) we need the following result on the stochastic integrable inequality.

**Lemma 8 (see [13]).**

Assume that functions *Y* ∈ *C*(*R*_+_ × *Ω*, *R*_+_) and *Z* ∈ *C*(*R*_+_ × *Ω*, *R*_+_) satisfy lim_*t*→*∞*_(*Z*(*t*)/*t*) = 0  a.s. If there is *T* > 0 such that

(34) $$\begin{array}{c} \ln Y\left(\;t\;\right)\geq \nu_{0}t-\nu \overset{t}{\underset{0}{\int }}Y\left(\;s\;\right)ds+Z\left(\;t\;\right)\text{ a.s.}, \end{array}$$

for all *t* ≥ *T*, then

(35) $$\begin{array}{c} \underset{t\to \infty }{lim\;inf}\frac{1}{t}\overset{t}{\underset{0}{\int }}Y\left(\;s\;\right)ds\geq \frac{\nu_{0}}{\nu }\text{ a.s.} \end{array}$$

## 3. Extinction of Disease

For the convenience of following statements, we denote

(36) S0=1−qdR+εΛdSdR+ε+pdR,S1=dIdR+ε+dRγdSdR+ε+pdR.

We further define a threshold value

(37) R~0=fS0g′0β+∑j=1lσ0jσ2jdI+γ−fS0g′02σ022dI+γ−σ222dI+γ.

**Theorem 9 .**

Assume that (**H**_1_) and (**H**_2_) hold. If one of the following conditions holds:

(a) ${\tilde{R}}_{0}<1$  and  *σ*_0_^2^*f*(*S*_0_)*g*′(0) ≤ *β* + ∑_*j*=1_^*l*^*σ*_0*j*_*σ*_2*j*_,
(b) *σ*_0_ > 0  and  (*β* + ∑_*j*=1_^*l*^*σ*_0*j*_*σ*_2*j*_)^2^/2*σ*_0_^2^ − (*d*_*I*_ + *γ* + (1/2)*σ*_2_^2^) < 0,

then, for any initial value (*S*(0), *I*(0), *R*(0)) ∈ *R*_+_^3^, one has

(38) $$\begin{array}{c} \underset{t\to \infty }{\mathrm{limsup}}\frac{\ln I\left(t\right)}{t}<0\text{ a.s.} \end{array}$$

That is, disease *I* is stochastically extinct exponentially with probability one. Moreover,

(39) limt→∞St=S0,limt→∞Rt=ΛqdS+pdSdR+ε+pdR  a.s.

**Proof**

Applying Itô's formula to model (6) leads to

(40) ln⁡It=ln⁡I0+∫0tfxsds+∑j=1lσ0j∫0tfSsgIsIsdBjs−σ2jBjt,

where *x* = (*S*, *I*) and

(41) fx=fSgIIβ+∑j=1lσ0jσ2j−dI+γ+σ222−12fSgII2σ02.

Assume that condition (b) holds. Since

(42) $$\begin{array}{c} f\left(\;x\left(\;t\;\right)\;\right)\leq \frac{{\left(\beta +\sum_{j=1}^{l}\sigma_{0j}\sigma_{2j}\right)}^{2}}{2\sigma_{0}^{2}}-\left(\;d_{I}+\gamma +\frac{1}{2}\sigma_{2}^{2}\;\right), \end{array}$$

then from (40)

(43) ln⁡Itt≤ln⁡I0t+β+∑j=1lσ0jσ2j22σ02−dI+γ+12σ22+∑j=1lσ0j1t∫0tfSsgIsIsdBjs−σ2j1t∫0tdBjs.

By Lemma 2, we have

(44) limt→∞1t∫0tSsgIsIsdBjs=0,limt→∞1t∫0tdBjs=0  a.s.,1≤j≤l.

Therefore,

(45) limsupt→∞⁡ln⁡Ittβ+∑j=1lσ0jσ2j22σ02−dI+γ+12σ22<0.

Assume that condition (a) holds. Choose constant *ϵ* > 0 such that *β* + ∑_*j*=1_^*l*^*σ*_0*j*_*σ*_2*j*_ ≥ *g*′(0)*f*(*ϵ*)*σ*_0_^2^. We compute that

(46) fx=fSgIIβ+∑j=1lσ0jσ2j−dI+γ+σ222−12fSgII2σ02=fSgIIβ+∑j=1lσ0jσ2j−dI+γ+σ222+12f2ϵg2II2σ02−12fS−fϵ2g2II2σ02−fSfϵg2II2σ02≤β+∑j=1lσ0jσ2jgII−fϵσ02g2II2fS−dI+γ+σ222+12fϵg′02σ02.

When *σ*_0_^2^ = 0, which implies *σ*_0*j*_ = 0  (1 ≤ *j* ≤ *l*), we have from (46)

(47) fxβfSgII−dI+γ+σ222≤βg′0fS−dI+γ+σ222.

Since

(48) fSfS0+fS−fS0=fS0+f′ξS−S0,

where *ξ* ∈ (*S*, *S*_0_), from (*H*_2_), we can obtain *f*′(*ξ*)(*S* − *S*_0_) ≤ *f*′(*S*_0_)(*S* − *S*_0_). Hence, we have

(49) $$\begin{array}{c} f\left(\;S\;\right)\leq f\left(\;S_{0}\;\right)+f^{\prime }\left(\;S_{0}\;\right)\left(\;S-S_{0}\;\right). \end{array}$$

According to (14), (40), and (49), we have

(50) ln⁡Itt≤ln⁡I0t+βg′01t∫0tfSsds−dI+γ+σ222+∑j=1lσ0j1t∫0tfSsgIsIsdBjs−σ2jBjtt=ln⁡I0t+βg′0fS0−βg′0·f′S0S1It+βf′S0g′0φt−dI+γ+σ222+∑j=1lσ0j1t∫0tfSsgIsIsdBjs−σ2j1t∫0tdBjs.

Hence, from (44) and Lemma 3, we finally have

(51) $$\begin{array}{c} \underset{t\to \infty }{\mathrm{limsup}}\frac{\ln I\left(t\right)}{t}\leq \beta f\left(\;S_{0}\;\right)g^{\prime }\left(\;0\;\right)-\left(\;d_{I}+\gamma +\frac{\sigma_{2}^{2}}{2}\;\right)\text{ a.s.} \end{array}$$

When *σ*_0_^2^ ≠ 0, from (40) and (46) we have

(52) ln⁡Itt≤ln⁡I0t+1t∫0tβ+∑j=1Nσ0jσ2jgIsIs−fϵσ02g2IsIs2fSsds+∑j=1Nσ0j1t∫0tfSsgIsIsdBjs−σ2jBjtt−dI+γ+σ222+12fϵg′02·σ02.

Define a function

(53) $$\begin{array}{c} F\left(\;u\;\right)=\left(\;\beta +\overset{l}{\underset{j=1}{\sum }}\sigma_{0j}\sigma_{2j}\;\right)u-f\left(\;\epsilon \;\right)\sigma_{0}^{2}u^{2}. \end{array}$$

Clearly, *F*(*u*) is a monotone increasing for *u* ∈ [0, (*β* + ∑_*j*=1_^*l*^*σ*_0*j*_*σ*_2*j*_)/2*f*(*ϵ*)*σ*_0_^2^] and monotone decreasing for *u* ∈ [(*β* + ∑_*j*=1_^*l*^*σ*_0*j*_*σ*_2*j*_)/2*f*(*ϵ*)*σ*_0_^2^, *∞*). With condition *β* + ∑_*j*=1_^*l*^*σ*_0*j*_*σ*_2*j*_ ≥ *g*′(0)*f*(*ϵ*)*σ*_0_^2^, that is, *g*(*I*)/*I* ≤ *g*′(0) ≤ (*β* + ∑_*j*=1_^*l*^*σ*_0*j*_*σ*_2*j*_)/2*f*(*ϵ*)*σ*_0_^2^, we have

(54) FgIIFg′0=β+∑j=1lσ0jσ2jg′0−fϵσ02g′02.

Hence, by (14) and (49), we have

(55) ln⁡Itt≤ln⁡I0t+g′0β+∑j=1lσ0jσ2j−fϵ·σ02g′01t∫0tfSsds+∑j=1lσ0j1t∫0tfSsgIsIsdBjs−σ2j1t∫0tdBjs−dI+γ+σ222+12g′0·fϵ2σ02≤ln⁡I0t+g′0β+∑j=1lσ0jσ2j−fϵσ02g′0fS0+f′S0φt−dI+γ+σ222+12g′0fϵ2σ02+∑j=1lσ0j1t∫0tSsgIsIsdBjs−σ2j1t∫0tdBjs.

Choose *ϵ* = *S*_0_; from (44) and Lemma 3, we finally have

(56) limsupt→∞⁡ln⁡Itt≤fS0g′0β+∑j=1lσ0jσ2j−dI+γ+σ222−12fS0g′02σ02  a.s.

From (45), (51), and (56), it follows that (38) holds.

Since lim_*t*→*∞*_*I*(*t*) = 0  a.s., by (14) of Lemma 3 and the last equation of (15), we further obtain

(57) limt→∞St=S0,limt→∞Rt=ΛqdS+pdSdR+ε+pdR  a.s.

This completes the proof.

**Remark 10 .**

Condition (b) in Theorem 9 can be rewritten in the following form:

(58) $$\begin{array}{c} \sigma_{0}^{2}>\frac{{\left(\beta +\sum_{j=1}^{l}\sigma_{0j}\sigma_{2j}\right)}^{2}}{2\left(d_{I}+\gamma +\left(1/2\right)\sigma_{2}^{2}\right)}. \end{array}$$

It is clear that

(59) fS0g′0β+∑j=1lσ0jσ2jdI+γ+1/2σ22−fS0g′02β+∑j=1lσ0jσ2j24dI+γ+1/2σ222≤1.

Therefore, when condition (b) holds, from (58) we also have

(60) R~0=fS0g′0β+∑j=1lσ0jσ2jdI+γ−fS0g′02σ022dI+γ−σ222dI+γ<fS0g′0β+∑j=1lσ0jσ2jdI+γ−fS0g′02β+∑j=1lσ0jσ2j24dI+γdI+γ+1/2σ22−σ222dI+γ=fS0g′0β+∑j=1lσ0jσ2jdI+γ+1/2σ22−fS0g′02β+∑j=1lσ0jσ2j24dI+γ+1/2σ222×dI+γ+1/2σ22dI+γ−σ222dI+γ≤1.

**Remark 11 .**

From Remark 10 above, we see that in Theorem 9 if condition (a) holds, then we directly have ${\tilde{R}}_{0}<1$, and if condition (b) holds, then we also have ${\tilde{R}}_{0}<1$. Therefore, an interesting open problem is whether we can establish the extinction of disease *I* with probability one for model (6) only when ${\tilde{R}}_{0}<1$.

## 4. Stochastic Persistence in the Mean

In this section, we discuss the stochastic persistence and permanence in the mean with probability one for model (6) only for the following two special cases: (1) *σ*_0*j*_ = 0  (1 ≤ *j* ≤ *l*) and (2) *σ*_1*j*_ = *σ*_2*j*_ = *σ*_3*j*_ = 0  (1 ≤ *j* ≤ *l*). Furthermore, we also assume that in model (6) function *f*(*S*) ≡ *S*.

### 4.1. Case *σ*_0*j*_ = 0  (1 ≤ *j* ≤ *l*)

When *f*(*S*) = *S* and *σ*_0*j*_ = 0  (1 ≤ *j* ≤ *l*) in model (6), we have

(61) $$\begin{array}{c} {\tilde{R}}_{0}=\frac{S_{0}g^{\prime }\left(0\right)\beta }{d_{I}+\gamma }-\frac{\sigma_{2}^{2}}{2\left(d_{I}+\gamma \right)}. \end{array}$$

**Theorem 12 .**

Assume that (**H**_1_) holds, *f*(*S*) = *S*, and *σ*_0*j*_ = 0  (1 ≤ *j* ≤ *l*). If ${\tilde{R}}_{0}>1$; then disease *I* in model (6) is stochastically persistent in the mean; that is,

(62) $$\begin{array}{c} \underset{t\to \infty }{lim\;inf}\frac{1}{t}\overset{t}{\underset{0}{\int }}I\left(\;r\;\right)dr>0\text{ a.s.} \end{array}$$

**Proof**

Let (*S*(*t*), *I*(*t*), *R*(*t*)) be any positive solution of model (6). Lemma 2 implies that there is a constant *M*_0_ > 0 such that *S*(*t*) + *I*(*t*) + *R*(*t*) ≤ *M*_0_  a.s. for all ≥0. Define a Lyapunov function

(63) $$\begin{array}{c} U\left(\;I\;\right)=\overset{I\left(t\right)}{\underset{I\left(0\right)}{\int }}\frac{1}{g\left(I\right)}dI. \end{array}$$

Using Itô's formula to model (6) leads to

(64) dUI=βS−dI+γIgI−σ222I2g2Ig′Idt−∑j=1lσ2jIgIdBjt=βS−dI+γIgI−1g′0−σ222I2g2Ig′I−1g′0−dI+γg′0+σ222g′0dt−∑j=1lσ2jIgIdBjt.

From (*H*_1_), which implies that *g*′(*I*) ≤ *g*(*I*)/*I* ≤ *g*′(0), we have

(65) I2g2Ig′I−1g′0=I2g2Ig′I−g′0+g′0I2g2I−1g′02≤g′0IgI+1g′0IgI−1g′0≤Mg′0+1IgI−1g′0,

where *M* = sup_0≤*I*≤*M*_0__{*I*/*g*(*I*)}. Since lim_*I*→0^+^_(*I*/*g*(*I*)) = 1/*g*′(0), then 0 < *M* < *∞*. Substituting (65) into (64) and then integrating from 0 to *t* ≥ 0, we get

(66) UIt≥1t∫0tβSr−dI+γ+σ222Mg′0+1·IrgIr−1g′0−dI+γg′0+σ222g′0dr−∑j=1l1tMjt,

where *M*_*j*_(*t*) = ∫_0_^*t*^*σ*_2*j*_(*I*(*r*)/*g*(*I*(*r*)))*dB*_*j*_(*r*). From Lemma 2, we have

(67) $$\begin{array}{c} \underset{t\to \infty }{lim}\frac{1}{t}M_{j}\left(\;t\;\right)=0\text{ a.s.},\;1\leq j\leq l. \end{array}$$

Define a function *G*(*I*) as follows. When *I* > 0, *G*(*I*) = *I*/*g*(*I*), and when *I* = 0, *G*(0) = lim_*I*→0_(*I*/*g*(*I*)) = 1/*g*′(0). Then *G*(*I*) is continuous for *I* ≥ 0 and differentiable for *I* > 0. Applying Lagrange's mean value theorem to *G*(*I*) − *G*(0), we have from (66)

(68) UIt≥1t∫0tβSr−dI+γ+σ222Mg′0+1·sup0≤I≤M0G′IIr−dI+γg′0+σ222g′0dr−∑j=1l1tMjt,

Substituting (14) into (68), it follows that

(69) UIt≥βS0−βS1+dI+γ+σ222Mg′0+1sup0≤I≤M0G′I·It−dI+γg′0+σ222g′0−∑j=1l1tMjt+βφt.

Since

(70) UIt1t∫I0ItIgI1IdI≤1t∫I0ItM1IdI=Mln⁡It−ln⁡I0t,

we have

(71) ln⁡Itt≥1MβS0−dI+γg′0−σ222g′0−1MβS1+dI+γ+σ222Mg′0+1sup0≤I≤M0G′I·It+Φt,

where

(72) $$\begin{array}{c} \Phi \left(\;t\;\right)=-\frac{1}{M}\overset{l}{\underset{j=1}{\sum }}\frac{1}{t}M_{j}\left(\;t\;\right)+\frac{\beta \varphi \left(t\right)}{M}+\frac{1}{t}\ln I\left(\;0\;\right). \end{array}$$

From (67) and Lemma 3 we have lim_*t*→*∞*_Φ(*t*) = 0. Finally, by Lemma 8, we obtain

(73) $$\begin{array}{c} \underset{t\to \infty }{lim\;inf}\frac{1}{t}\overset{t}{\underset{0}{\int }}I\left(\;r\;\right)dr\geq I^{*}, \end{array}$$

where

(74) $$\begin{array}{c} I^{*}=\frac{\left(d_{I}+\gamma \right)\left({\tilde{R}}_{0}-1\right)}{\left(\beta S_{1}+\left(d_{I}+\gamma +\left(1/2\right)\sigma_{2}^{2}\left(Mg^{\prime }\left(0\right)+1\right)\right)\sup_{0\leq I\leq M_{0}}\left\{G^{\prime }\left(I\right)\right\}\right)g^{\prime }\left(0\right)}. \end{array}$$

This completes the proof.

**Remark 13 .**

In the proof of Theorem 12, we easily see that three constants *M*_0_, *M* = sup_0≤*I*≤*M*_0__{*I*/*g*(*I*)}, and *I*^*∗*^ given in (74) are dependent on every solution (*S*(*t*), *I*(*t*), *R*(*t*)) of model (6). This shows that in Theorem 12 we only obtain the stochastic persistence in the mean of the disease.

### 4.2. Case *σ*_1*j*_ = *σ*_2*j*_ = *σ*_3*j*_ = 0  (1 ≤ *j* ≤ *l*)

When *f*(*S*) = *S* and *σ*_1*j*_ = *σ*_2*j*_ = *σ*_3*j*_ = 0  (1 ≤ *j* ≤ *l*) in model (6), we have

(75) $$\begin{array}{c} {\tilde{R}}_{0}=\frac{\beta S_{0}g^{\prime }\left(0\right)}{d_{I}+\gamma }-\frac{S_{0}^{2}{\left(g^{\prime }\left(0\right)\right)}^{2}\sigma_{0}^{2}}{2\left(d_{I}+\gamma \right)}. \end{array}$$

In order to obtain the stochastic permanence in the mean with probability one for model (6), we need to introduce a new threshold value

(76) $$\begin{array}{c} {\bar{R}}_{0}=\frac{\beta S_{0}g^{\prime }\left(0\right)}{d_{I}+\gamma }-\frac{{\bar{S}}^{2}{\left(g^{\prime }\left(0\right)\right)}^{2}\sigma_{0}^{2}}{2\left(d_{I}+\gamma \right)}. \end{array}$$

Obviously, we have ${\bar{R}}_{0}\leq {\tilde{R}}_{0}$.

**Theorem 14 .**

Assume that (**H**_1_) holds, *f*(*S*) = *S*, and *σ*_1*j*_ = *σ*_2*j*_ = *σ*_3*j*_ = 0  (1 ≤ *j* ≤ *l*). If ${\bar{R}}_{0}>1$, then disease *I* in model (6) is stochastically permanent in the mean, that is,

(77) lim inft→∞1t∫0tIrdr≥dI+γR¯0−1βS1+dI+γmax0≤I≤S¯⁡ G′Ig′0  a.s.,

where function *G*(*I*) is defined in Theorem 12 above.

**Proof**

Let *U*(*I*) = ∫_*I*(0)_^*I*(*t*)^(1/*g*(*I*))*dI*; using Itô's formula to model (6) and (18) leads to

(78) dUI=βS−dI+γIgI−σ022S2g′Idt−∑j=1lSσ0jdBjt≥βS−dI+γIgI−1g′0−σ02S¯2g′02+dI+γg′0dt−∑j=1lSσ0jdBjt.

Similarly to above proof of Theorem 12, we have

(79) UIt≥1t∫0tβSr−dI+γmax0≤I≤S¯⁡ G′IIr−σ02S¯2g′02+dI+γg′0dr−∑j=1l∫0tSrσ0jdBjr.

Substituting (14) into (79) yields

(80) UIt≥βS0−βS1+dI+γmax0≤I≤S¯⁡ G′I1t∫0tIrdr−σ02S¯2g′02+dI+γg′0−∑j=1l1t∫0tSrσ0jdBjr+βφt.

Since by (18)

(81) $$\begin{array}{c} \frac{U\left(I\right)}{t}\leq \frac{1}{t}\overset{I\left(t\right)}{\underset{I\left(0\right)}{\int }}\frac{\bar{S}}{g\left(\bar{S}\right)}\frac{1}{I}dI\leq \frac{\bar{S}\left(\ln\bar{S}-\ln I\left(0\right)\right)}{g\left(\bar{S}\right)t}, \end{array}$$

(80) can be rewritten as

(82) 1t∫0tIrdr≥dI+γR¯0−1+g′0ΦtβS1+dI+γmax0≤I≤S¯⁡ G′Ig′0,

where

(83) Φt=−∑j=1l1t∫0tSrσ0jdBjr+βφt−S¯ln⁡S¯−ln⁡I0gS¯t.

By Lemmas 2 and 3, it follows that lim_*t*→*∞*_Φ(*t*) = 0. Therefore, taking *t* → *∞* in (82) it follows that (77) holds. This completes the proof.

Using Lemma 5, we can establish the following result which shows that ${\tilde{R}}_{0}$ can be a threshold value for the stochastic permanence of disease *I* in the mean for a more special case of model (6): *d*_*S*_ = *d*_*R*_ and *d*_*I*_ = *d*_*S*_ + *α* with constant *α* ≥ 0.

**Theorem 15 .**

Assume that (**H**_1_) holds, *f*(*S*) = *S*, *σ*_1*j*_ = *σ*_2*j*_ = *σ*_3*j*_ = 0  (1 ≤ *j* ≤ *l*), *d*_*S*_ = *d*_*R*_, and *d*_*I*_ = *d*_*S*_ + *α* with constant *α* ≥ 0. If ${\tilde{R}}_{0}>1$; then disease *I* in model (6) is stochastically permanent in the mean; that is,

(84) lim inft→∞1t∫0tIrdr≥dS+α+γR~0−1βS1+dI+γmax0≤I≤S¯⁡ G′I+M0g′0  a.s.,

where function *G*(*I*) is defined in above Theorem 12 and

(85) M0=61+α2dS2+γ2dS+ε2+p2dS+ε+p2+pα2dS2dS+ε+p2+pγ2dS+ε2dS+ε+p2ΛdS.

**Proof**

Firstly, when *d*_*S*_ = *d*_*R*_ and *d*_*I*_ = *d*_*S*_ + *α*, then threshold value ${\tilde{R}}_{0}$ becomes

(86) $$\begin{array}{c} {\tilde{R}}_{0}=\frac{\beta S_{0}g^{\prime }\left(0\right)}{d_{S}+\alpha +\gamma }-\frac{S_{0}^{2}{\left(g^{\prime }\left(0\right)\right)}^{2}\sigma_{0}^{2}}{2\left(d_{S}+\alpha +\gamma \right)}, \end{array}$$

where *S*_0_ = Λ[(1 − *q*)*d*_*S*_ + *ε*]/*d*_*S*_(*d*_*S*_ + *ε* + *p*).

Let *U*(*I*) = ∫_*I*(0)_^*I*(*t*)^(1/*g*(*I*))*dI*; similarly to above proof of Theorem 12, we have

(87) dUI≥βS−dS+α+γmax0≤I≤S¯ G′II−12σ02S2g′0−dS+α+γg′0dt−∑j=1lSσ0jdBjt.

Since *S*^2^ = *S*_0_^2^ + 2*S*_0_(*S* − *S*_0_)+(*S* − *S*_0_)^2^, we further have

(88) UIt≥βSt−dS+α+γmax0≤I≤S¯ G′IIt−12σ02S02g′0−σ02S0g′0St−S0−dS+α+γg′0−12σ02g′0St−S02−∑j=1l1t∫0tSσ0jdBjs.

Substituting (14) and (21) of Lemma 5 into (88), using inequality (*a* + *b*)^2^ ≤ 2(*a*^2^ + *b*^2^), it follows that

(89) UIt≥dS+α+γg′0R~0−1−βS1+dS+α+γmax0≤I≤S¯ G′IIt+β−σ02S0g′0φt−σ02g′0H2t+G2t−∑j=1l1t∫0tSσ0jdBjs.

From expression (22) of *H*(*t*), we easily have lim_*t*→*∞*_〈*H*^2^(*t*)〉 = 0. By (18), without loss of generality, we can assume that *S*(*t*) + *I*(*t*) + *R*(*t*) ≤ Λ/*d*_*S*_  a.s. for all *t* ≥ 0. Hence,

(90) G2t≤6I2t+α2∫0te−dSt−sIsds2+γ2∫0te−dS+εt−sIsds2+p2∫0te−dS+ε+pt−sIsds2+pα2·∫0te−dS+ε+pt−s∫0se−dSs−uIudu ds2+pγ2∫0te−dS+ε+pt−s·∫0se−dS+εs−uIudu ds2≤6ΛdSIt+α2ΛdS2∫0te−dSt−sIsds+γ2ΛdSdS+ε·∫0te−dS+εt−sIsds+p2ΛdSdS+ε+p·∫0te−dS+ε+pt−sIsds+pα2λdS2dS+ε+p·∫0te−dS+ε+pt−s∫0se−dSs−uIudu ds+pγ2ΛdSdS+εdS+ε+p∫0te−dS+ε+pt−s·∫0se−dS+εs−uIudu ds.

By computing, we obtain

(91) 1t∫0t∫0se−dSs−uIudu ds≤1dSIt,1t∫0t∫0se−dS+εs−uIudu ds≤1dS+εIt,1t∫0t∫0se−dS+ε+ps−uIudu ds≤1dS+ε+pIt,1t∫0t∫0se−dS+ε+ps−u∫0ue−dSu−vIvdv du ds≤1dSdS+ε+pIt,1t∫0t∫0se−dS+ε+ps−u∫0ue−dS+εu−vIvdv du ds≤1dS+εdS+ε+pIt.

Therefore, we finally have

(92) $$\begin{array}{c} \langle \;G^{2}\left(\;t\;\right)\;\rangle \leq M_{0}\langle \;I\left(\;t\;\right)\;\rangle . \end{array}$$

From (81), (89), and (92) we further obtain

(93) 1t∫0tIrdr≥dS+α+γR~0−1+g′0ΦtβS1+dI+γmax0≤I≤S¯⁡ G′I+M0g′0,

where

(94) Φt=−∑j=1l1t∫0tSrσ0jdBjr+β−σ02S0g′0φt−σ02g′0H2t−S¯ln⁡S¯−ln⁡I0gS¯t.

By Lemmas 2 and 3, it follows that lim_*t*→*∞*_Φ(*t*) = 0. Therefore, taking *t* → *∞* in (93) it follows that (84) holds. This completes the proof.

## 5. Stationary Distribution

In this section, we discuss the stationary distribution of model (6) by using Lyapunov function method. We firstly define the diffusion matrix *A*(*x*) = *h*(*x*)*h*^*T*^(*x*), where *x* = (*S*, *I*, *R*),

(95) hx=h11xh12x⋯h1lxh21xh22x⋯h2lxh31xh32x⋯h3lx,h1jx=−fSgIσ0j−fSσ1j,h2jx=fSgIσ0j−Iσ2j,h3jx=−Rσ3j.

Furthermore, we denote by *a*_*ii*_(*x*)  (*i* = 1,2, 3) the diagonal elements of matrix *A*(*x*). We have *a*_*ii*_(*x*) = ∑_*j*=1_^*l*^*h*_*ij*_^2^(*x*).

**Theorem 16 .**

Assume that (**H**_1_) holds, *f*(*S*) = *S*, and there is a constant *ρ* > 0 such that *a*_*ii*_(*x*) > *ρ*, for any *x* ∈ *R*_+_^3^ and *i* = 1,2, 3, *γ* > *p*, *d*_*I*_ > *d*_*S*_, and *γ*(*d*_*S*_ + *d*_*R*_) > *p*(*d*_*I*_ + *d*_*R*_). If *R*_0_ > 1 and

(96) dS+pγdS+dR−pdI+dRγ−pε+dS−C1S∗2∧dI+γγdS+dR−pdI+dRγ−pε+dI−C2I∗2∧dI−dSdR+εγ−p+dR−C3R∗2>dS+dIγ−pε+dS+p+dI+γγdS+dR−pdI+dRI∗σ22εβgI∗γ−p+C1S∗2+C2I∗2+C3R∗2,

where

(97) C1=2dS+dIγ−pε+dS+p+dI+γγdS+dR−pdI+dRI∗g′02σ02εβgI∗γ−p+2γdS+dR−pdI+dRσ12γ−pε+σ2,C2=2γdS+dR−pdI+dRσ22γ−pε+σ2,C3=dI−dSσ32γ−p+σ2

and (*S*^*∗*^, *I*^*∗*^, *R*^*∗*^) is the unique endemic equilibrium of model (2), then model (6) has a unique stationary distribution.

**Proof**

We here use the Lyapunov function method to prove this theorem. The proof given here is similar to Theorem 5.1 in [11]. But, due to nonlinear function *g*(*I*), the Lyapunov function structured in the following is different from that given in [11].

By Lemma 7, it suffices to find a nonnegative Lyapunov function *V*(*x*) and compact set *K* ⊂ *R*_+_^3^ such that *LV*(*x*)≤−*C* for some *C* > 0 and *x* ∈ *R*_+_^3^/*K*.

Denote *x* = (*S*, *I*, *R*) ∈ *R*_+_^3^. Define the function

(98) $$\begin{array}{c} V_{1}\left(\;x\;\right)=\frac{1}{2}{\left(\;R-R^{*}\;\right)}^{2}. \end{array}$$

Calculating *LV*_1_(*x*), we have

(99) LV1x=R−R∗·pS−S∗+γI−I∗−dR+εR−R∗+12σ32R2≤−dR+ε−σ32R−R∗2+pS−S∗R−R∗+γI−I∗R−R∗+σ32R∗2.

Define the function

(100) $$\begin{array}{c} V_{2}\left(\;x\;\right)=I-I^{*}-I^{*}\ln\frac{I}{I^{*}}. \end{array}$$

Calculating *LV*_2_(*x*), we have

(101) LV2x=1−I∗IβSgI−dI+γI+I∗2·∑j=1lSgIIσ0j−σ2j2=I−I∗·βSgII−gI∗I∗+βgI∗I∗S−S∗+12I∗σ22+12I∗σ02S2g2II2−∑j=1lI∗SgIIσ0jσ2j≤βgI∗I∗S−S∗I−I∗+12I∗σ22+12·I∗σ02g′02S2−∑j=1lI∗SgIIσ0jσ2j≤β·gI∗I∗S−S∗I−I∗+2I∗σ02g′02·S−S∗2+2I∗σ02g′02S∗2+I∗σ22.

Define the function

(102) $$\begin{array}{c} V_{3}\left(\;x\;\right)=\frac{1}{2}{\left(\;S+I-S^{*}-I^{*}\;\right)}^{2}. \end{array}$$

Calculating *LV*_3_(*x*), we get

(103) LV3x=S+I−S∗−I∗−dS+pS−S∗+εR−R∗−dI+γI−I∗+12σ12S2+12·σ22I2+∑j=1lSIσ1jσ2j≤−dS+p−2σ12S−S∗2−dI+γ−2σ22I−I∗2+εS−S∗R−R∗−dS+p+dI+γS−S∗I−I∗+εI−I∗R−R∗+2σ12S∗2+2σ22I∗2.

Define the function

(104) $$\begin{array}{c} V_{4}\left(\;x\;\right)=\frac{1}{2}{\left(\;S+I+R-S^{*}-I^{*}-R^{*}\;\right)}^{2}. \end{array}$$

Calculating *LV*_4_(*x*), we get

(105) LV4x=S+I+R−S∗−I∗−R∗·Λ−dSS−dII−dRR+12·∑j=1lSσ1j+Iσ2j+Rσ3j2≤−dSS−S∗2−dII−I∗2−dRR−R∗2−dS+dI·S−S∗I−I∗−dS+dRS−S∗R−R∗−dI+dRI−I∗R−R∗+12σ12+σ22+σ32·S2+I2+R2≤−dS−σ2S−S∗2−dI−σ2I−I∗2−dR−σ2R−R∗2−dS+dIS−S∗I−I∗−dS+dRS−S∗·R−R∗−dI+dRI−I∗R−R∗+σ2S∗2+I∗2+R∗2.

Define the Lyapunov function for model (6) as follows:

(106) Vx=dS+dIγ−pε+dS+p+dI+γγdS+dR−pdI+dRI∗εβgI∗γ−pV2x+dI−dSγ−pV1x+γdS+dR−pdI+dRγ−pεV3x+V4x.

Then from (99), (101), (103), and (105) we have

(107) LVx−dS+pγdS+dR−pdI+dRγ−pε+dS−C1S−S∗2−dI+γγdS+dR−pdI+dRγ−pε+dI−C2I−I∗2−dI−dSdR+εγ−p+dR−C3R−R∗2+C1S∗2+C2I∗2+C3R∗2+dS+dIγ−pε+dS+p+dI+γγdS+dR−pdI+dRI∗σ22εβgI∗γ−p.

If condition (96) holds, then the surface

(108) dS+pγdS+dR−pdI+dRγ−pε+dS−C1S−S∗2+dI+γγdS+dR−pdI+dRγ−pε+dI−C2I−I∗2+dI−dSdR+εγ−p+dR−C3R−R∗2=dS+dIγ−pε+dS+p+dI+γγdS+dR−pdI+dRI∗σ22εβgI∗γ−p+C1S∗2+C2I∗2+C3R∗2

lies in the interior of *R*_+_^3^. Hence, we can easily obtain that there exists a constant *C* > 0 and a compact set *K* of *R*_+_^3^ such that, for any *x* ∈ *R*_+_^3^/*K*,

(109) $$\begin{array}{c} LV\left(\;x\;\right)\leq -C. \end{array}$$

Therefore, model (6) has a unique stationary distribution. This completes the proof.

**Remark 17 .**

In fact, the variances of errors usually should be small enough to justify their validity of real data; otherwise, the data may not be considered as a good one. It is clear that when *σ*_*ij*_ are very small, condition (96) is always satisfied.

## 6. Numerical Examples

To verify the theoretical results in this paper, we next give numerical simulations of model (6).

Throughout the following numerical simulations, we choose *l* = 2 and *g*(*I*) = *I*/(1 + *ωI*^2^), where *ω* is a positive constant. It is easy to verify that assumption (*H*_1_) holds. By Milstein's higher-order method [29, 30], we drive the corresponding discretization equations of model (6):

(110) Si+1=Si+1−qΛ−βfSiIi1+ωIi2−dSi+PSi+εRiΔt−fSiIi1+ωIi2∑j=1lσ0jξjiΔt+12σ0j2ξji2−1Δt−Si∑j=1lσ1jξjiΔt+12σ1j2ξji2−1Δt,Ii+1=Ii+βfSiIi1+ωIi2−dI+γIiΔt+fSiIi1+ωIi2·∑j=1lσ0jξjiΔt+12σ0j2ξji2−1Δt−Ii∑j=1lσ2jξjiΔt+12σ2j2ξji2−1Δt,Ri+1=Ri+qΛ+pSi+γIi−dR+εRiΔt−Ri∑j=1lσ3jξjiΔt+12σ3j2ξji2−1Δt.

Here, *ξ*_*ji*_  (*i* = 1,2,…, *j* = 1,…, *l*) are *N*(0,1)-distributed independent Gaussian random variables and Δ*t* > 0 is time increment.

**Example 1 .**

**Figure 1:**
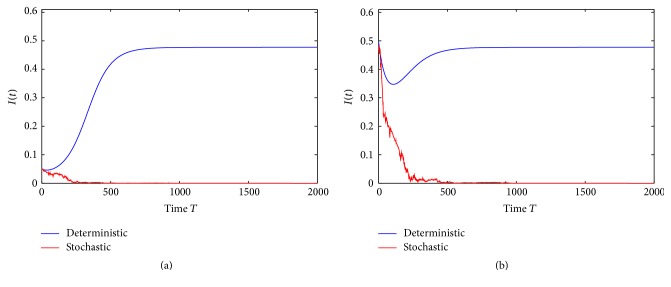
The path of *I*(*t*) for the stochastic model (6) with parameters in Example 1, compared to the corresponding deterministic model. (a) is trajectories of the solution *I*(*t*) with the initial value *I*(0) = 0.05 and (b) with the initial value *I*(0) = 0.5. The disease of model (6) is extinct with probability one.

In model (6), we take *f*(*S*) = *S*/(1 + 0.2*S*), Λ = 1.85, *q* = 0.52, *β* = 0.52, *p* = 0.24, *ε* = 0.2, *γ* = 0.3, *ω* = 2, *d*_*S*_ = 0.4, *d*_*I*_ = 0.21, *d*_*R*_ = 0.3, *σ*_01_ = 0.15, *σ*_02_ = 0.99, *σ*_11_ = 0.23, *σ*_12_ = 0.17, *σ*_21_ = 0.14, *σ*_22_ = 0.72, *σ*_31_ = 0.47, and *σ*_32_ = 0.93. By computing, we obtain ${\tilde{R}}_{0}=0.8939<1$, *σ*_0_^2^*f*(*S*_0_)*g*′(0)−(*β* + ∑_*j*=1_^2^*σ*_0*j*_*σ*_2*j*_) = 0.3442 > 0, and (*β* + ∑_*j*=1_^2^*σ*_0*j*_*σ*_2*j*_)^2^/2*σ*_0_^2^ − (*d*_*I*_ + *γ* + (1/2)*σ*_2_^2^) = 0.005 > 0. This shows that conditions (a) and (b) of Theorem 9 do not hold. The numerical simulations (see Figure 1) suggest that disease *I*(*t*) of model (6) is still stochastically extinct with probability one. Therefore, as an improvement of Theorem 9, we have the following interesting conjecture.

**Conjecture 2 .**

Assume (*H*_1_) holds. The disease *I*(*t*) in model (6) is stochastically extinct with probability one only when ${\tilde{R}}_{0}<1$ holds.

**Example 3 .**

**Figure 2:**
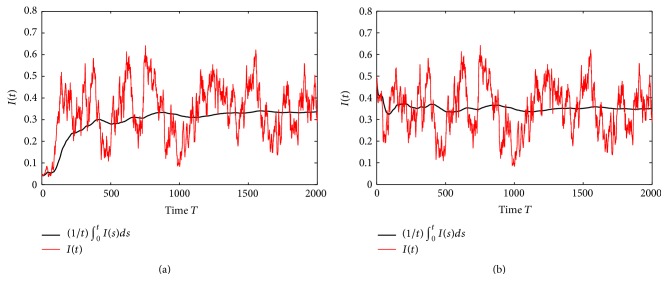
The paths of *I*(*t*) and (1/*t*)∫_0_^*t*^*I*(*s*)*ds* for the stochastic model (6) with parameters in Example 3, (a) with the initial value *I*(0) = 0.05 and (b) with the initial value *I*(0) = 0.5.

In model (6), we take *f*(*S*) = *S*/(1 + 1.5*S*), Λ = 3, *q* = 0.2, *β* = 2.1, *p* = 0.3, *ε* = 0.8, *γ* = 0.1, *ω* = 2, *d*_*S*_ = 0.5, *d*_*I*_ = 0.8, *d*_*R*_ = 0.4, *σ*_01_ = 0.8, *σ*_02_ = 1.2, *σ*_11_ = 0.3, *σ*_12_ = 0.75, *σ*_21_ = 0.45, *σ*_22_ = 0.8, *σ*_31_ = 0.8, and *σ*_32_ = 0.3. By computing, we obtain ${\tilde{R}}_{0}=1.3554>1.$ From the numerical simulations given in Figure 2, it is shown that disease *I*(*t*) of model (6) is not only stochastically persistent in the mean but also stochastically persistent with probability one. Therefore, as an improvement of Theorem 12, we have the following interesting conjecture.

**Conjecture 4 .**

Assume (*H*_1_) holds. The disease *I*(*t*) in model (6) is stochastically persistent in the mean only when ${\tilde{R}}_{0}>1$.

**Example 5 .**

**Figure 3:**
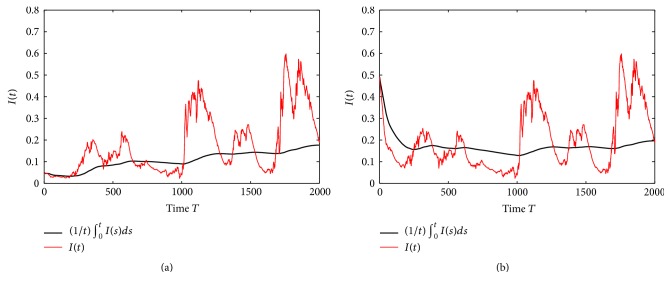
The paths of *I*(*t*) and (1/*t*)∫_0_^*t*^*I*(*s*)*ds* for the stochastic model (6) with parameters in Example 5, (a) with the initial value *I*(0) = 0.05 and (b) with the initial value *I*(0) = 0.5.

In model (6), we take *f*(*S*) = *S*/(1 + 0.1*S*), Λ = 1.2, *q* = 0.5, *β* = 1.5, *p* = 0.9, *ε* = 1.1, *γ* = 0.9, *ω* = 2, *d*_*S*_ = 0.6, *d*_*I*_ = 0.35, *d*_*R*_ = 0.4, *σ*_01_ = 0.4, *σ*_02_ = 0.2, *σ*_11_ = 0.1, *σ*_12_ = 0.45, *σ*_21_ = 0.2, *σ*_22_ = 0.1, *σ*_31_ = 0.2, and *σ*_32_ = 0.3. By computing, we obtain ${\bar{R}}_{0}=0.8687<1$ and ${\tilde{R}}_{0}=1.2931>1.$ The numerical simulations given in Figure 3 show that disease *I*(*t*) of model (6) is still stochastically permanent in the mean. Therefore, combining Theorem 12 and Theorem 14, we can obtain the following interesting conjecture about the stochastic permanence in the mean of disease *I*(*t*).

**Conjecture 6 .**

Assume (*H*_1_) holds. The disease *I*(*t*) in model (6) is stochastically permanent in the mean only when ${\tilde{R}}_{0}>1$.

**Example 7 .**

In model (6), we take *f*(*S*) = *S*, Λ = 0.67, *q* = 0.02, *β* = 1.7, *p* = 0.05, *ε* = 3, *γ* = 0.99, *ω* = 4, *d*_*S*_ = 0.29, *d*_*I*_ = 0.53, *d*_*R*_ = 0.39, *σ*_01_ = 0.025, *σ*_02_ = 0.02, *σ*_11_ = 0.0121, *σ*_12_ = 0.01, *σ*_21_ = 0, *σ*_22_ = 0, *σ*_31_ = 0.02, and *σ*_32_ = 0.01. By computing, we obtain that the basic reproduction number for deterministic model (2) is *R*_0_ = 2.5279 > 1 and the unique endemic equilibrium of model (2) is (*S*^*∗*^, *I*^*∗*^, *R*^*∗*^) = (1.4230,0.3845,0.1372). Furthermore, we can verify that there is a constant *ρ* > 0 such that *a*_*ii*_(*x*) > *ρ* for any *x* ∈ *R*_+_^3^  (*i* = 1,2, 3), *d*_*I*_  −  *d*_*S*_ = 0.24 > 0, *γ*  −  *p* = 0.94 > 0, *γ*(*d*_*S*_ + *d*_*R*_) − *p*(*d*_*I*_ + *d*_*R*_) = 0.6272 > 0, and

(111) dS+pγdS+dR−pdI+dRγ−pε+dS−C1S∗2∧dI+γγdS+dR−pdI+dRγ−pε+dI−C2I∗2∧dI−dSdR+εγ−p+dR−C3R∗2−dS+dIγ−pε+dS+p+dI+γγdS+dR−pdI+dRI∗σ22εβgI∗γ−p+C1S∗2+C2I∗2+C3R∗2=0.0147>0.

**Figure 4:**
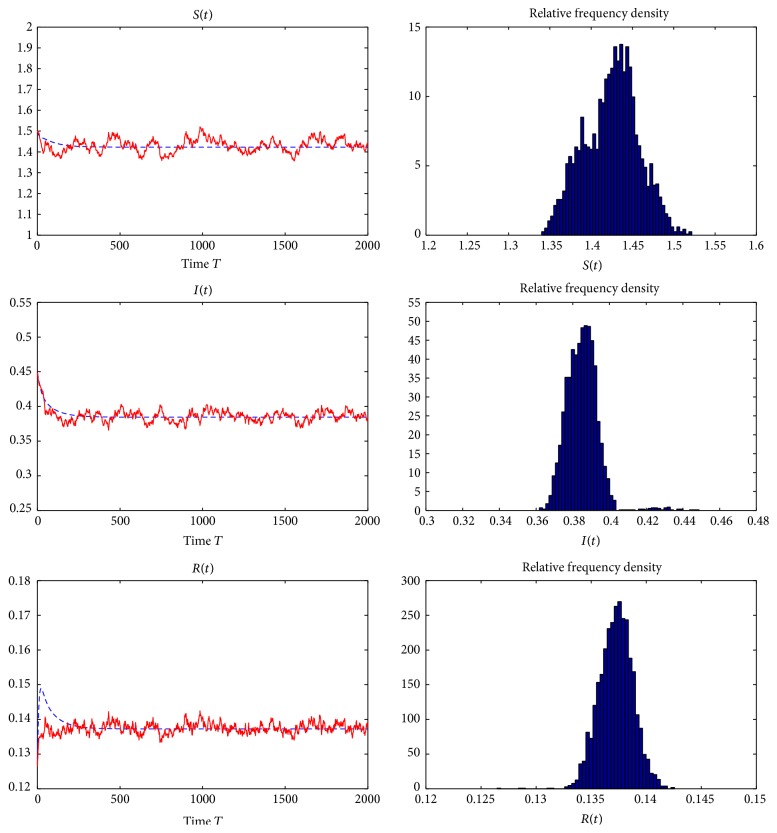
The solution of stochastic model (6) and its histogram with parameters in Example 7.

That is, all conditions in Theorem 16 are satisfied. The stationary distributions about the susceptible, infected, and removed individuals obtained through the numerical simulations are reported in Figure 4, which shows that after some initial transients the population densities fluctuate around the deterministic steady-state values *S*^*∗*^ = 1.4230, *I*^*∗*^ = 0.3845, and *R*^*∗*^ = 0.1372.

**Example 8 .**

In model (6), we take *f*(*S*) = *S*/(1 + 0.4*S*), Λ = 2.5, *q* = 0.5, *β* = 1.4, *p* = 0.7, *ε* = 0.9, *γ* = 0.51, *ω* = 1.89, *d*_*S*_ = 0.7, *d*_*I*_ = 0.45, *d*_*R*_ = 0.58, *σ*_01_ = 0.4, *σ*_02_ = 0.2, *σ*_11_ = 0.21, *σ*_12_ = 0.1, *σ*_21_ = 0.1, *σ*_22_ = 0.24, *σ*_31_ = 0.2, and *σ*_32_ = 0.1. By computing, we obtain that the basic reproduction number for deterministic model (2) is *R*_0_ = 1.6484 > 1 and the unique endemic equilibrium of model (2) is (*S*^*∗*^, *I*^*∗*^, *R*^*∗*^) = (1.7242,0.5082,1.8352). Furthermore, we can verify that there is not a constant *ρ* > 0 such that *a*_*ii*_(*x*) > *ρ* for any *x* ∈ *R*_+_^3^ and *i* = 1,2, 3, *d*_*I*_ − *d*_*S*_ = −0.25 < 0, *γ* − *p* = −0.19 < 0, *γ*(*d*_*S*_ + *d*_*R*_) − *p*(*d*_*I*_ + *d*_*R*_) = −0.0682 < 0 and

(112) dS+pγdS+dR−pdI+dRγ−pε+dS−C1S∗2∧dI+γγdS+dR−pdI+dRγ−pε+dI−C2I∗2∧dI−dSdR+εγ−p+dR−C3R∗2−dS+dIγ−pε+dS+p+dI+γγdS+dR−pdI+dRI∗σ22εβgI∗γ−p+C1S∗2+C2I∗2+C3R∗2=−5.5051<0.

**Figure 5:**
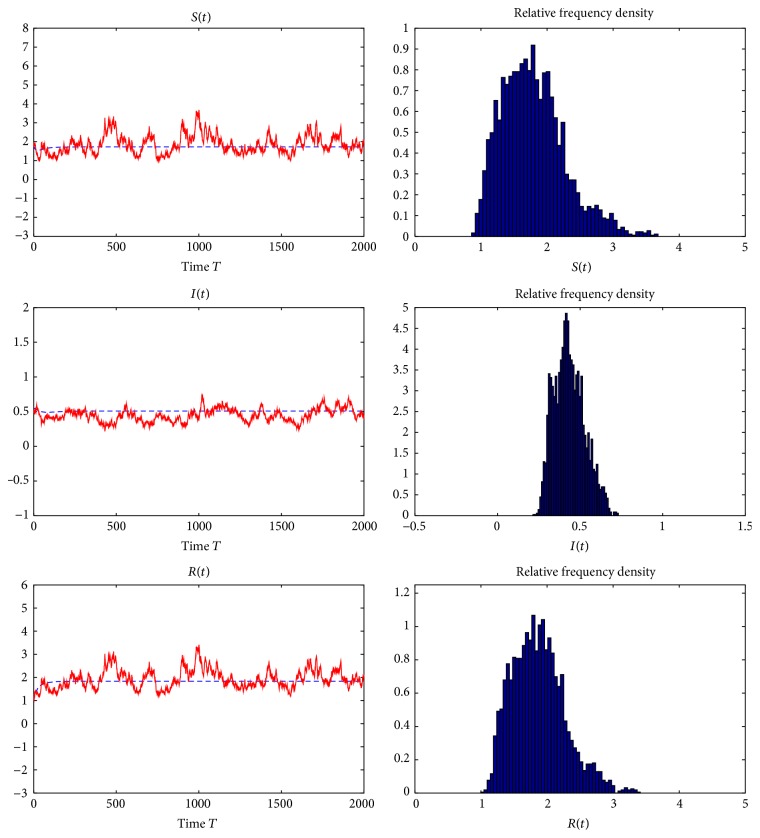
The solution of stochastic model (6) and its histogram with parameters in Example 8.

That is, the conditions in Theorem 16 are not satisfied. However, we obtain that threshold value ${\tilde{R}}_{0}=2.7192>1.$ The numerical simulations given in Figure 5 show the stationary distributions about the susceptible, infected, and removed individuals. Therefore, we can obtain the following interesting conjecture about the stationary distribution for model (6), as described in the conclusion part.

**Conjecture 9 .**

Assume (*H*_1_) holds. Model (6) has a unique stationary distribution only when ${\tilde{R}}_{0}>1$.

## 7. Conclusion

In this paper, as an extension of the results given in [11, 25], we investigated the dynamical behaviors for a stochastic SIRS epidemic model (6) with nonlinear incidence and vaccination. In model (6), the disease transmission coefficient *β* and the removal rates *d*_*S*_, *d*_*I*_, and *d*_*R*_ are affected by noise. Some new basic properties of model (6) are found in Lemmas 2, 3, and 5. Applying these lemmas, we established a series of new threshold value criteria on the stochastic extinction, persistence in the mean, and permanence in the mean of the disease with probability one. Furthermore, by using the Lyapunov function method, a sufficient condition on the existence of unique stationary distribution for model (6) is also obtained.

The stochastic persistence and permanence in the mean of the disease for model (6) are established in this paper only for the special cases: *f*(*S*) ≡ *S* and (1) *σ*_0*j*_ = 0  (1 ≤ *j* ≤ *l*) or (2) *σ*_1*j*_ = *σ*_2*j*_ = *σ*_3*j*_ = 0  (1 ≤ *j* ≤ *l*). However, for the general model (6), particularly, *f*(*S*) ≠ *S* and (*σ*_1*j*_, *σ*_2*j*_, *σ*_3*j*_)≠(0,0, 0)  (1 ≤ *j* ≤ *l*), whether we also can establish similar results still is an interesting open problem.

In fact, under the above case, from the proofs of Theorems 12 and 14, we can see that an important question is to deal with terms *βf*(*S*(*t*)) and *f*^2^(*S*(*t*))*g*′(*I*(*t*)). If we may get

(113) βfSt≥βfS0+v1St−S0  a.s.,f2Stg′It≤f2S0g′0+v2St−S0  a.s.,

where *v*_1_ and *v*_2_ are two positive constants; then the following perfect result may be established.

Assume that (*H*_1_) holds. If ${\tilde{R}}_{0}>1$, then disease *I* in model (6) is stochastically persistent in the mean; that is,

(114) $$\begin{array}{c} \underset{t\to \infty }{lim\;inf}\frac{1}{t}\overset{t}{\underset{0}{\int }}I\left(\;r\;\right)dr>0\text{ a.s.} \end{array}$$

Another important open problem is about the existence of stationary distribution of model (6), that is, whether we can establish a similar result as in Theorem 16 when *f*(*S*) is a nonlinear function. The best perfect result on the stationary distribution is to prove that model (6) possesses a unique stationary distribution only when threshold value ${\tilde{R}}_{0}>1$. But this is a very difficult open problem.

However, the numerical examples given in Section 6 propose some affirmative answer for above open problems.
